# The Burden of Skin Cancers in Saudi Arabia Through 2011-2022

**DOI:** 10.7759/cureus.45052

**Published:** 2023-09-11

**Authors:** Mansour N AlOtaibi, Abdullah S Basfar, Amjad M Jawhari, Essam S Alzahrani, Mohammed A Althomali, Adnan E Alhindi, Samir S Alam, Daifallah M Al Aboud, Ahmed S Abdel-Moneim

**Affiliations:** 1 College of Medicine, Taif University, Taif, SAU; 2 Department of Histopathology and Cytology, King AbdulAziz Specialist Hospital, Taif, SAU; 3 Department of Internal Medicine, Taif University, Taif, SAU; 4 Department of Microbiology, College of Medicine, Taif University, Taif, SAU

**Keywords:** saudi arabia, hiv, human herpes virus type 8, kaposi sarcoma, skin cancers

## Abstract

Introduction

Skin cancers are classified into melanoma and non-melanoma or keratinocyte cancers. No recent data are found about the epidemiology of skin cancers in Saudi Arabia. The current study aims to determine the burden of skin cancer in the last 11 years from 2011 to 2022.

Methods

Patients who were diagnosed with any type of skin cancer were enrolled in the study. The diagnosis was conducted based on histopathology and immunohistochemistry. Different variables like age, type of cancer, type of lesions, and treatment approach used were measured.

Results

A total of 91 patients were diagnosed with skin cancers during the study period. The head and neck were the most common sites for skin cancers. Only 4/91 cases reported invasive melanoma. Both squamous cell carcinoma (SCC) (34/91) and basal cell carcinoma (BCC) (28/91) were found to be the most reported skin cancers. Other cancers including mycosis fungoides (MF) (10/91), Kaposi’s sarcoma (6/91), and dermatofibrosarcoma protuberans (DFSP) (5/91) were also detected. The rest of the detected tumors were rarely detected. Aggressive CD4+/CD4+/CD8+ MF was more prevalent than CD3+/CD4+/CD8- MF cancer cases. CD34+ /S100- DFSP cancers were evident in most of the DFSP cases. Human herpes virus 8 was detected in all Kaposi’s sarcoma cases and all of them were HIV-confirmed cases. Surgical treatment was the most frequently used approach to treat skin cancers, followed by phototherapy (9.9%), surgical/radiotherapy (5.5%), surgical/chemotherapy (4.4%), chemotherapy (3.3%), and then chemoradiotherapy immunotherapy (1.1%).

Conclusion

The incidences of SCC and BCC are relatively high in comparison to other types of skin cancers with the surgical intervention being most frequently used.

## Introduction

Although skin cancers develop mainly in sun-exposed areas, on rare occasions, they may develop in unexposed areas of the skin. Non-melanoma skin cancers (NMSCs) are the most common types of cancer followed by melanoma. Melanoma is the most aggressive skin cancer derived from melanocytes in the basal layer of the epidermis [1]. Melanoma results from mutagens as exposure to UV rays may activate oncogenes with subsequent uncontrolled proliferation of melanocytes and cancer and can be developed in any area of the body either in already existing moles or in normal skin [1].

NMSCs include basal skin carcinoma (BCC), squamous cell carcinoma (SCC), basosquamous cell carcinoma (BSCC), mycosis fungoides (MF), dermatofibrosarcoma protuberans (DFSP), and Kaposi sarcoma (KS) among others [2]. Both SCC and BCC account for 95% of all cases [3]. Skin cancers are rarely fatal; however, they constitute a considerable economic burden to health [4].

BCC arises from the basal layer of the epidermis and its appendages. It also results from long-term exposure to UV radiation which induces cell mutations and appears most often on sun-exposed regions such as the nose, ears, face, and backs of hands, although it can appear elsewhere on the body [5]. It grows slowly and rarely metastasizes. Sun exposure, fair complexion, and light eyes are all risk factors for BCC and other types of skin cancers, but they also include chronic arsenic exposure, ionizing radiation, and basal cell nevus syndrome. The three main subtypes of BCC are superficial, nodular, and morphea forms. SCC develops from keratinocytes in the epidermis or adnexal structures like eccrine glands or pilosebaceous units [5]. The risk factors of SCC are the same as those of BCC, but they also include chronic inflammatory response to laceration, scar, burn, ulcer, or other type of skin injury, and epidermolysis bullosa syndromes. SCC is usually characterized by rough, erythematous papules, plaques, and nodules with well-defined borders and crusting. Ulceration, pigmentation, erythema, scaling, or hyperkeratosis may be also present in the lesions [6].

BSCC is defined as any tumor that has areas of BCC and a transition zone but does not change into SCC, or more broadly as any tumor that has characteristics of both BCC and SCC. BSCC is an aggressive type of BCC, with a nonspecific clinical presentation and a biopsy required to confirm the diagnosis. The head and neck are the most common sites for lesions [6].

MF is the most common cutaneous T-cell lymphoma, which constitutes about 60-80% of all those reported [7,8]. It is characterized by erythematous patches or plaques that are progressive, with scaling and atrophy, usually on sun-protected parts, with abnormal lymphocytic infiltrates of the epidermis (epidermotropism) under the microscope [9]. Most MF express CD3/CD4 but rarely express CD8 [10]. CD3+/CD4+/CD8+ MF was found to be more aggressive than CD3+/CD4+/CD8- MF cancers [11]. Meanwhile, DFSP is a rare soft-tissue tumor that appears at the age of 20-50 years with a CD34 tumor marker present in the majority of such types of cancer [12].

KS is a very rare cancer that arises from the skin's blood vessels. KS appears in the form of red or purple patches on any part of the skin and it can also be developed in the mucous membranes. This type of cancer is caused by human herpes virus 8 and develops only in immunocompromised people such as people with AIDS. It also may affect patients treated with immunosuppressive therapy for a long period such as people who have undergone organ transplantation [13,14]. Vascular cancers express both CD34 and CD31. The former is less specific as a tumor marker; however, the latter is more specific to KS although it showed less sensitivity than CD34 [15,16].

A definitive diagnosis of skin malignancy is usually made with a skin biopsy and histopathologic examination [17]. Although there are some studies reporting skin cancer in Saudi Arabia, there are no reports on the incidence of skin cancer in Taif, a high-altitude city in Saudi Arabia. Therefore, this study was conducted to fill this gap and determine the incidence of major skin cancers. To our knowledge, no estimate of the incidence of skin cancers has been published to date.

## Materials and methods

Ethical approval

Ethical approvals were obtained from both the Directorate of Health Affairs - Taif, Research and Studies Department (No. 717 on 01/08/2022) and Research and Ethics Committee-Western Region, Medical Services General Directorate, Ministry of Defense (No. 2022-639 on 29/06/2022).

Patients

A retrospective multicenter observational study at Taif City, Saudi Arabia, was conducted between January 2011 and December 2022. Three centers were included in the current study including Al-Hada Military Hospital, King Abdulaziz Hospital, and King Faisal Hospital. The patients who were diagnosed with skin cancers were extracted from the databases of these centers. Eligible patients showed definite histopathologic and/or immunostaining diagnosis of skin cancer. Patients' data including age, sex, site of the lesion, and year of the initial diagnosis were obtained from the database in an anonymized approach. Skin cancers were clustered based on histopathologic diagnosis.

Histopathology

Skin biopsies were fixed in 10% neutral buffered formalin. After trimming, the fixed tissues were washed, dehydrated in the ascending concentrations of ethyl alcohol, and cleared in xylene, before paraffin embedding. A rotatory microtome was used to section the formalin-fixed paraffin-embedded (FFPE) sections into 5 μm thickness. The tissue sections were then mounted on glass slides and stained with hematoxylin and eosin according to [18]. Selective histopathological sections were used to be imaged.

Immunohistochemistry

Immunostaining was used to identify tumor markers in different types of FFPE sections of skin cancers. Monoclonal antibodies used include human melanoma black antibody 45 (HMB-45) for melanoma, CD3, CD8, CD20, and CD30 for MF, human herpes virus 8 Mab and CD31 for KS, and CD34 and S100 for DFSP. Ventana Discovery Ultra Auto Stainer (Roche Diagnostics, Indianapolis, USA) protocol for immunohistochemistry was adopted following the manufacturer’s instructions. Briefly, paraffin sections were deparaffinized by heating slides for 4 min at 72°C and treated with EZ prep solution (Roche Ventana, Tucson, AZ, USA). Slides were then incubated at 95°C for 56 min in Cell Conditioning Solution 1 (CC1, Roche Ventana) to retrieve the antigen. Primary antibodies were then applied to the slides for 56 min at 36°C, followed by incubation with DISCOVERY OmniMap anti-Mouse HRP kit (Roche Ventana) for 16 min. The DISCOVERY ChromoMap DAB detection kit (Roche Ventana) was used as the detection system. Hematoxylin II was used as a counter-staining.

Statistical analysis

A descriptive analysis using crosstab was conducted to calculate the numbers and percentages of different variables. Chi-square was used to screen the significance of different variables using SPSS version 16.0 (IBM, Armonk, NY, USA).

## Results

Distribution of cancers over the years

A total of 91 cancer cases were reported including 51 from King Abdul-Aziz Hospital, seven from King Faisal Hospital, and 33 from Al-Hada Military Hospital. Melanoma was detected in four patients only (4/91, 4.4%). Both SCC (34/91, 37.4%) and BCC (28/91, 30.8%) were found to be the most reported skin cancers. A considerable number of other cancers including MF (10/91, 11%), KS (6/91, 6.6%), and DFSP (5/91, 5.5%) were also detected. Angiosarcoma (1/91, 1.1%), eccrine poroma (1/91, 1.1%), neuroendocrine tumor (1/91, 1.1%), and giant cell tumor (1/91, 1.1% ) were found rarely. Although not statistically significant, however, there is a marked increase in the total reported skin cancers from 2019 to 2021 with the highest number reported in 2021 (24/91 cases, 26.4%) in comparison to other years (Table 1). This increase in 2021 was specifically reported in SCC (n: 8), BCC (n: 8), and MF (n: 5, 5.5%) (Table 1). Both age and sex significantly affect the development of the lesion based on the Wilcoxon signed ranks test (P<0.001).

**Table 1:** Distribution of the reported skin cancer over years Data are expressed as numbers of cases.

Lesion	Year												Total
	2011	2012	2013	2014	2015	2016	2017	2018	2019	2020	2021	2022	
Melanoma	0	0	0	0	0	1	0	2	0	0	0	1	4
Squamous cell carcinoma	1	0	0	2	5	3	2	1	5	5	8	2	34
Basal cell carcinoma	0	1	0	0	0	3	2	2	8	3	8	1	28
Mycosis fungoides	0	1	0	0	0	0	0	0	2	2	5	0	10
Kaposi sarcoma	0	0	1	0	0	0	0	1	0	2	1	1	6
Dermatofibrosarcoma protuberans	0	1	0	0	1	0	0	0	1	0	2	0	5
Angiosarcoma	0	0	0	0	1	0	0	0	0	0	0	0	1
Eccrine poroma	0	0	0	0	0	0	0	0	0	0	0	1	1
Neuroendocrine tumor	0	0	0	0	0	0	1	0	0	0	0	0	1
Giant cell tumor	0	0	0	0	0	0	0	0	1	0	0	0	1
Total	1	3	1	2	7	7	5	6	17	12	24	6	91

Melanoma

**Figure 1:**
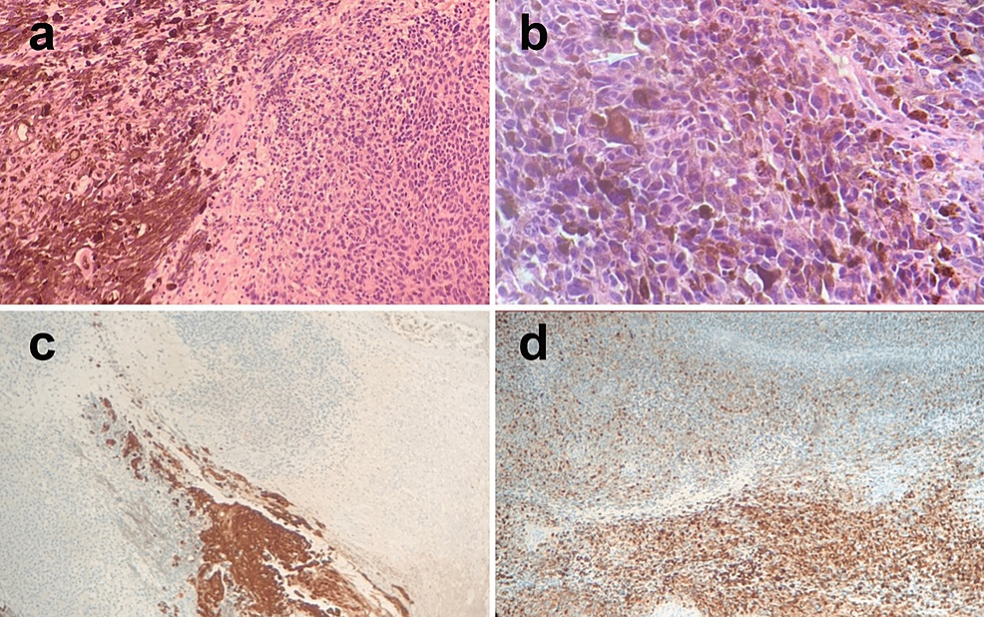
Histopathology of an incisional biopsy of melanoma presented as a chronic ulcerated mass in the right foot (upper middle and lower heel). a) Skin pigmented tumor distributed throughout the dermis with H& E staining with 20× magnification. b) H&E staining with 40× magnification showed polygonal and spindle cells with nuclear pleomorphism, prominent nucleoli, and intranuclear inclusion. c) Immunohistochemistry using HMB-45 showed positive staining in a large number of cells ×10. d) Immunohistochemistry using HMB-45 showed positive staining ×20. HMB-45, human melanoma black antibody 45.

Only four out of the total 91 skin cancer cases (4.4%) were found to be melanoma. They included two cases (2.2%) in the lower limb, one (1.1%) in the head and neck, and one (1.1%) in the upper limb (Table 2). Two cases (2.2%) were detected in 2016, one in 2018, and one in 2022. Melanoma was detected in two males and two females (Table 3). The lesions were detected over a wide age range: 1 (1.1%) in 20-30 years, 1 (1.1%) in 51-60, 1 (1.1%) in 61-70, and the last case in a patient over 70 years old (Table 3). Tumor cells extend up to all margins. Sections show skin pigmented tumors distributed throughout the dermis; they show large numbers of brown-colored melanocytes (pigmented skin tumors) distributed throughout the dermis and subcutaneous tissues (Figure 1a). The tumor was composed of polygonal to spindle cells with nuclear pleomorphism, prominent nucleoli, and intranuclear inclusion (Figure 1b). Immunohistochemistry using HMB-45 revealed positive immunostaining in melanoma cells (Figure 1c, 1d) and negative to CK5/6 Mab.

**Table 2:** The distribution of the skin cancer based on the pathological classification and the site of the skin lesions. Chi-square P<0.001, R=0.303, P<0.004. Data are expressed as numbers and percentages [N(%)]. ^a^Solitary lesion in the head. ^b^Two cases showed solitary lesion in the left leg and two cases showed multiple lesions; one case showed multiple nodules in both legs and thigh, another case showed multiple lesions in the left knee, right foot, as well as right hand. ^c^Multiple papules and plaques on the left forearm.

Site of the lesion	Type of skin cancer										Total
	Melanoma	Squamous cell carcinoma	Basal cell carcinoma	Mycosis fungoides	Kaposi sarcoma	Dermatofibrosarcoma protuberans	Angiosarcoma	Eccrine poroma	Neuroendocrine tumor	Giant cell tumor	
Head and neck	1 (1.1%)	19 (20.9%)	25 (27.5%)	0	1 (1.1%)^a^	2 (2.2%)	0 (0.0%)	0 (0.0%)	0 (0.0%)	0 (0.0%)	48 (52.7%)
Lower limb	2 (2.2%)	6 (6.6%)	1 (1.1%)	1 (1.1%)	4 (4.4%) ^b^	1 (1.1%)	1 (1.1%)	0 (0.0%)	0 (0.0%)	0 (0.0%)	16 (17.6%)
Trunk	0 (0.0%)	6 (6.6%)	1 (1.1%)	2 (2.2%)	0 (0.0%)	1 (1.1%)	0 (0.0%)	0 (0.0%)	1 (1.1%)	0 (0.0%)	11 (12.1%)
Upper limb	1 (1.1%)	1 (1.1%)	1 (1.1%)	1 (1.1%)	1 (1.1%)^c^	0 (0.0%)	0 (0.0%)	1 (1.1%)	0 (0.0%)	1 (1.1%)	7 (7.7%)
Widespread	0 (0.0%)	0 (0.0%)	0 (0.0%)	6 (6.6%)	0 (0.0%)	0 (0.0%)	0 (0.0%)	0 (0.0%)	0 (0.0%)	0 (0.0%)	6 (6.6%)
Buttocks	0 (0.0%)	2 (2.2%)	0 (0.0%)	0 (0.0%)	0 (0.0%)	1 (1.1%)	0 (0.0%)	0 (0.0%)	0 (0.0%)	0 (0.0%)	3 (3.3%)
Cumulative	4 (4.4%)	34 (37.4%)	28 (30.8%)	10 (11.0%)	6 (6.6%)	5 (5.5%)	1 (1.1%)	1 (1.1%)	1 (1.1%)	1 (1.1%)	91 (100.0%)

**Table 3:** The age and sex of the patients with different types of skin cancers. Chi-square for sex P<0.016, age P<0.009. Data are expressed as numbers and percentages [N(%)].

Variable	Type of skin cancer										Total
	Melanoma	Squamous cell carcinoma	Basal cell carcinoma	Mycosis fungoides	Kaposi sarcoma	Dermatofibrosarcoma protuberans	Angiosarcoma	Eccrine poroma	Neuroendocrine tumor	Giant cell tumor	
Sex											
Male	2 (2.2%)	21 (23.1%)	20 (22.0%)	4 (4.4%)	4 (4.4%)	4 (4.4%)	1 (1.1%)	0 (0.0%)	1 (1.1%)	0 (0.0%)	57 (62.6%)
Female	2 (2.2%)	13 (14.3%)	8 (8.8%)	6 (6.6%)	2 (2.2%)	1 (1.1%)	0 (0.0%)	1 (1.1%)	0 (0.0%)	1 (1.1%)	34 (37.4%)
Age											
Less than 20	0 (0%)	1 (1.1%)	0 (0%)	1 (1.1%)	0 (0%)	0 (0%)	0 (0%)	0 (0%)	0 (0%)	0 (0%)	2 (2.2%)
20-30	1 (1.1%)	2 (2.2%)	0 (0%)	2 (2.2%)	0 (0%)	2 (2.2%)	0 (0%)	0 (0%)	0 (0%)	0 (0%)	7 (7.7%)
31-40	0 (0%)	2 (2.2%)	1 (1.1%)	0 (0%)	0 (0%)	2 (2.2%)	0 (0%)	1 (1.1%)	0 (0%)	1 (1.1%)	7 (7.7%)
41-50	0 (0%)	6 (6.6%)	3 (3.3%)	5 (5.5%)	0 (0%)	1 (1.1%)	0 (0%)	0 (0%)	0 (0%)	0 (0%)	15 (16.5%)
51-60	1 (1.1%)	5 (5.5%)	5 (5.5%)	2 (2.2%)	0 (0%)	0 (0%)	0 (0%)	0 (0%)	0 (0%)	0 (0%)	13 (14.3%)
61-70	1 (1.1%)	6 (6.6%)	6 (6.6%)	0 (0%)	0 (0%)	0 (0%)	0 (0%)	0 (0%)	0 (0%)	0 (0%)	13 (14.3%)
Above 70	1 (1.1%)	12 (13.2%)	13 (14.3%)	0 (0%)	6 (6.6%)	0 (0%)	1 (1.1%)	0 (0%)	1 (1.1%)	0 (0%)	34 (37.4%)
Cumulative	4 (4.4%)	34 (37.4%)	28 (30.8%)	10 (11%)	6 (6.6%)	5 (5.5%)	1 (1.1%)	1 (1.1%)	1 (1.1%)	1 (1.1%)	91 (100%)

Squamous cell carcinoma

SCC was the most commonly detected skin cancer in the current study; it was detected in 34 out of the total 91 skin cancer cases (37.4%). More than half of the cases, 19/34 (55.9%), were reported in the head and neck, followed by lower limb (6/34, 17.6%) and trunk (6/34, 17.6%). Only two cases were detected in the buttock and a single case in the upper limb (Table 2). There was no significant variation with the distribution of sex but there was a highly significant variation of the SCC among different age groups (P<0.009) where the highest incidence was reported in patients above 70 years old (12/34 cases, 35.3%) followed by 40-70 years (six cases in the age range of 41-50 years, five cases in the age range of 51-60 years, and six cases in the age range of 61-70 years) (Table 3).

**Figure 2:**
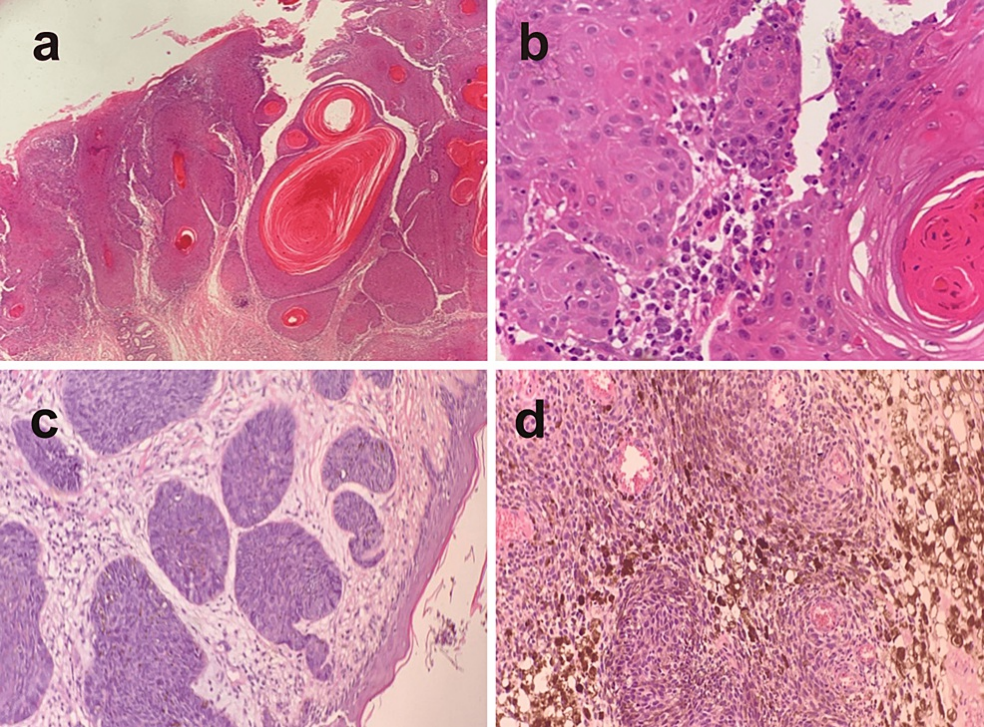
Histopathological pictures of squamous and basal cell carcinoma of skin cancer a) Squamous cell carcinoma showing cell nests of squamous epithelial cells (H&E 20x). b) Squamous cell carcinoma showing stromal desmoplastic response (H&E 40x). c) Basal cell carcinoma revealed that the dermis is deeply infiltrated with islands of malignant cells (H&E 20x). d) Basal cell carcinoma cells possess large, hyperchromatic, oval nuclei and little cytoplasm. Melanocytes appear uniform with no invasion of the blood vessels (H&E 40x).

Histopathology revealed well-differentiated SCC (Figure 2). Cells appear in cell nests of the squamous epithelial cells arising from the epidermis and extending into the dermis (Figure 2a). Tumor cells showed a stromal desmoplastic response. The malignant cells are often large with abundant cytoplasm and a large nucleus with visible variable keratinization (Figure 2b).

Basal cell carcinoma

As in the SCC, BCC was mostly found in the head and neck (25/28 cases, 89.3%), while a single case was found in the lower and upper limbs as well as the trunk (Table 2). Although not statistically significant, males showed a higher rate of incidence of BCC in comparison to females since 20/28 (71.4%) and 8/28 (28.6%) cases showed BCC in males and females, respectively. Age was found to be a significant factor in the development of BCC (P<0.009) since 13/28 cases were detected in patients above 70 years old. This was followed by 61-70 years (six cases) and 51-60 years (five cases) (Table 3). Histopathology sections revealed that the dermis is deeply infiltrated with islands of malignant cells (Figure 2c). The cells possess large, hyperchromatic, oval nuclei and little cytoplasm. Melanocytes appear uniform with no invasion of the blood vessels (Figure 2d). In addition, an infiltration of lymphocytes and fibroplasia among the tumor masses were detected.

Mycosis fungoides

Most of the cases (6/10, 60%) detected with MF showed widespread lesions all over the body, and the remaining cases were detected in the lower limb (one case), upper limb (one case), and two cases in the trunk (Table 2). This type of skin cancer was detected in four males and six females and over an age range of less than 20 years to 60 years with the highest incidence in 41-50 years old (Table 3). Most of the cases showed positive CD3+/CD4 immunostaining (Table 4). The cases that showed widespread lesions all over the body showed CD+ positive immunostaining (Table 4). A single case showed CD4- reaction and positive reaction to both CD20 and CD30 with unknown CD8 reactivity (Table 4).

**Table 4:** Immunohistochemistry using different tissue biomarkers in mycosis fungoides skin cancers ^a^CD: cluster of differentiation. ^b^Few scattered large cells. ​​​​​​​^c^NP: not performed.

Case number	CD3^a^	CD4	CD8	CD20	CD30
1	+^b^	+	+	-	NP^c ^
2	-	-	NP	+	+
3	NP	+	-	-	NP
4	+	+	-	-	NP
5	+	+	-	-	NP
6	+	+	+	NP	+
7	+	+	+		+
8	+	+	+	-	NP
9	+	+	+	-	NP
10	+	+	+	-	NP

**Figure 3:**
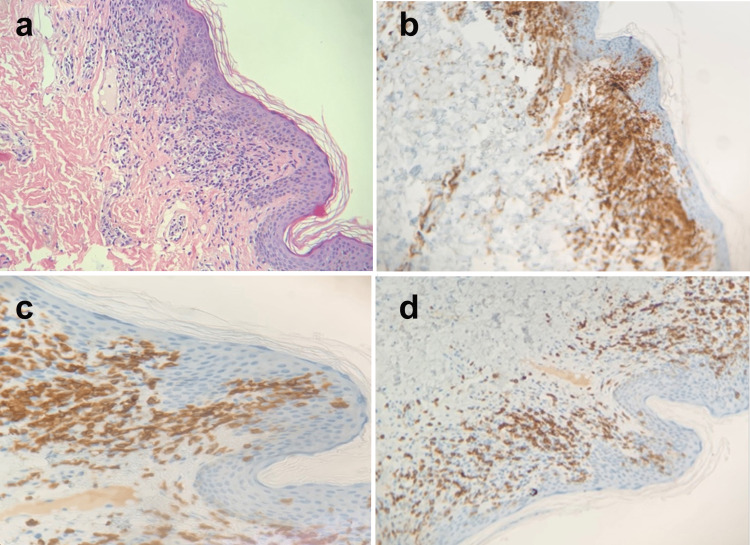
Histopathology and immunohistochemistry of abdominal skin biopsy with mycosis fungoides. a) Intercellular edema and diffuse infiltration of a huge number of lymphocytes in the dermis regarded as a fungal infection. b) Positive immunohistochemistry for CD3. c) Positive immunohistochemistry for CD4. d) Positive immunohistochemistry for CD8.

Most of the cases were treated with phototherapy with a single case treated by a combination of chemoradiotherapy and immunotherapy (Table 5). One case was reported in 2012, two in 2019, and two in 2020, while most of the cases were reported in 2021 (5/10 cases). Microscopic examination revealed intercellular edema and diffuse infiltration of a huge number of lymphocytes in the dermis regarded as a fungal infection (Figure 3a). Immunohistochemistry revealed that the tumor cells were positive for CD3 (Figure 3b), CD4 (Figure 3c), and CD8 (Figure 3d) while weak to negative for CD20 (Figure 3e).

**Table 5:** The treatment approach adopted in different types of skin cancers. Chi-square P<0.001. *Intralesional chemotherapy. Data are expressed as numbers and percentages [N(%)].

Treatment regimen	Type of skin cancer										Total
	Melanoma	Squamous cell carcinoma	Basal cell carcinoma	Mycosis fungoides	Kaposi sarcoma	Dermatofibrosarcoma protuberans	Angiosarcoma	Eccrine poroma	Neuroendocrine tumor	Giant cell tumor	
Surgical	2 (2.2%)	28 (30.8%)	27 (29.7%)	0 (0%)	4 (4.4%)	4 (4.4%)	1 (1.1%)	1 (1.1%)	1 (1.1%)	1 (1.1%)	69 (75.8%)
Phototherapy	0 (0%)	0 (0%)	0 (0%)	9 (9.9%)	0 (0%)	0 (0%)	0 (0%)	0 (0%)	0 (0%)	0 (0%)	9 (9.9%)
Surgical and radiotherapy	1 (1.1%)	3 (3.3%)	0 (0%)	0 (0%)	0 (0%)	1 (1.1%)	0 (0%)	0 (0%)	0 (0%)	0 (0%)	5 (5.5%)
Surgical and chemotherapy	0 (0%)	1 (1.1%)	1 (1.1%)	0 (0%)	2 (2.2%)*	0 (0%)	0 (0%)	0 (0%)	0 (0%)	0 (0%)	4 (4.4%)
Chemoradiotherapy	1 (1.1%)	2 (2.2%)	0 (0%)	0 (0%)	0 (0%)	0 (0%)	0 (0%)	0 (0%)	0 (0%)	0 (0%)	3 (3.3%)
Chemoradiotherapy immunotherapy	0 (0%)	0 (0%)	0 (0%)	1 (1.1%)	0 (0%)	0 (0%)	0 (0%)	0 (0%)	0 (0%)	0 (0%)	1 (1.1%)
Cumulative	4 (4.44%)	34 (37.4%)	28 (30.8%)	10 (11.0%)	6 (6.6%)	5 (5.5%)	1 (1.1%)	1 (1.1%)	1 (1.1%)	1 (1.1%)	91 (100%)

Kaposi’s sarcoma

**Figure 4:**
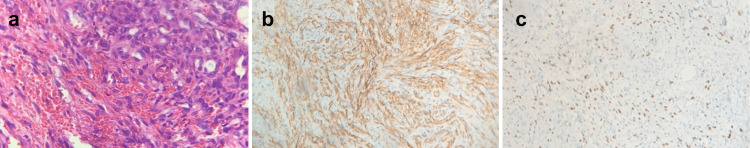
Histopathology and immunohistochemistry of Kaposi sarcoma lesion in the leg. a) H&E staining revealed multiple dilated vascular channels and spindle cell proliferation with mild nuclear atypia and extravasated red blood cells (40×). b). Positive IHC using Mab against HHV-8 (20×). c) IHC revealed positive using Mab against CD31 (20×). IHC, immunohistochemistry.

A total of six out of 91 skin tumors (6.6%) were found to be KS. All of them were detected to be positive for HIV. Four cases presented with a lesion in the lower limb while one showed a lesion in the head and the other a lesion in the trunk (Table 2). All reported cases were above 70 years old, four were males and two were females (Table 3). Four cases were treated with surgical intervention, while two cases were treated with both surgical and intralesional chemotherapy (Table 4). One case in 2013, one in 2018, two in 2020, one in 2021, and one in 2022. Histopathology revealed multiple dilated vascular channels dissecting through dermal collagen, spindle cell proliferation with mild nuclear atypia and mitotic activity and extravasated red blood cells (Figure 4a). Immunostaining revealed positive human herpes virus 8 (Figure 4b) and also CD31 (JC70/A) (Figure 4c).

Dermatofibrosarcoma protuberans

**Figure 5:**
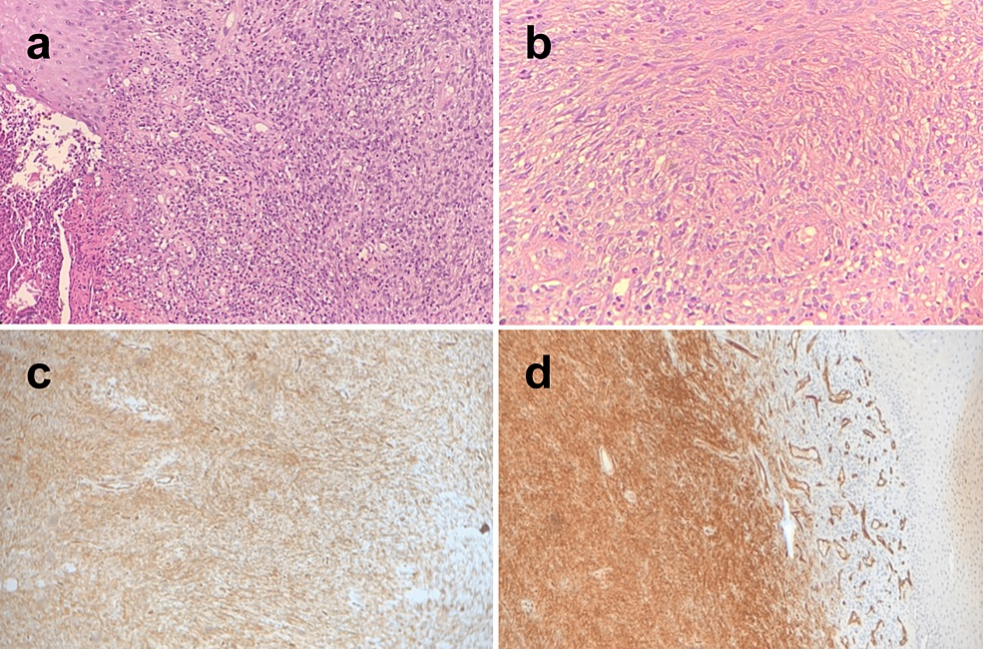
Dermatofibrosarcoma protuberans after incisional biopsy of fungating mass at the upper chest wall. a) Histopathology of a dermal tumor composed of spindled fibroblast-like cells. b) The tumor cells arranged in short fascicles and whorls. c) Positive immunostaining of CD34 20×. d) c) Intensive positive immunostaining of CD34 20×.

Five cases of DFSP were reported (5/91, 5.5%), which included two in the head and neck, one in each of the lower limb, upper limb, and buttock (Table 2). This type of cancer was reported in four males and one female in the relatively young age range (20-50 years old). Two cases in 20-30 years old, two in 31-40, and a single case in 41-50 years old (Table 3). The histopathology section showed an ulcerated epidermis with a dermal tumor composed of spindled fibroblast-like cells with moderate atypia and rare mitosis (Figure 5a). The tumor cells were arranged in short fascicles and whorls. There is a mild chronic inflammatory cell infiltrate of lymphocytes, neutrophils, and aggregates of foamy histiocytes (Figure 5a, 5b). Positive immunostaining of CD34 antigen was evident in four of five cases and was detected in many areas suggestive of fibro sarcomatous transformation (Table 4, Figure 5c, 5d). Meanwhile, S100 was negative in 4/5 cases and not performed in the fifth case (data not shown).

Other tumors

Three cases of eccrine poroma (female upper limb), neuroendocrine (male trunk), and giant cell tumors (female upper limb) were detected.

Treatment

Two out of the four melanoma cases (50%) were treated surgically while combined surgical and radiotherapy or chemoradiotherapy were used in the other two cases. Most of the cases of SCC were treated surgically (28/34, 82.3%) while three cases were treated with both surgery and radiotherapy, one case with surgery and chemotherapy, and two cases treated only with chemoradiotherapy (Table 5). As in SCC, surgical interference was the used treatment in most cases of BCC (27/28 cases, 96.4%) with only one case treated with both surgery and chemotherapy (Table 5). For KS, four cases were treated with surgical intervention only including two cases that showed a solitary lesion in the left leg and two cases that showed multiple lesions. One case showed multiple nodules in both legs and thigh, and the other showed multiple papules and plaques on the left forearm. Two cases were treated with both surgical intervention and intralesional chemotherapy, including one case that showed a solitary lesion in the head and the other case that showed multiple lesions in the left knee, right foot, and right hand (Tables 2, 5). Four cases of DFSP were treated with surgical intervention while the fifth case was treated with a combination of both surgical intervention and radiotherapy (Table 5). Three cases of eccrine poroma (female upper limb), neuroendocrine tumor (male trunk), and giant cell tumor (female upper limb) were treated by surgical intervention (Table 5).

## Discussion

Melanoma is the most serious case of skin cancer and constitutes the 13th and 15th most common cancer in men and women, respectively, overall. It is reported in one of every five skin cancer cases. In 2020, about 325,000 new cases were diagnosed with melanoma worldwide with 57,000 deaths [19]. HMB-45 expression is used to differentiate benign from malignant melanocytic tumors since it is indicative of early and metastatic melanoma [20]. Monoclonal antibody against this marker interacts with cancer cells but not with normal adult melanocytes or with nevus intradermal cells [21,22]. Immunostaining against the HMB-45 marker, although specific [20], showed variable sensitivities (69-93%) [23]. In the current study, four cases of melanoma were detected. HMB-45 immunostaining was positive in all melanoma cases that showed high intensity of positive cells. Melanoma was also detected in a previous study in Taif that was conducted between 1992 and 2001 and found 13 cases of melanoma among 104 skin cancer cases [24]. It was also reported in old studies in Al Madinah, Jeddah, Dharan, Qassim, and Riyadh [2,24,25-28].

NMSCs include a variety of skin cancer types. SCC and BCC are the dominant types of all NMSCs with the latter being more prevalent than the former [29]. There is a gender variation in the development of NMSC. Males were found to be more susceptible to developing NMSC than females [30]. In the early diagnosis, SCC and BCC possess good prognoses with very low mortality rates reported in both types [31]. On the other hand, and in agreement with other studies in Saudi Arabia, BCC is considered the most common skin cancer followed by SCC [2,24,25-27]. In contrast, in a study in northern Saudi Arabia (Asir), SCC was found more common in comparison to BCC [28]. Globally, although BCC followed by SCC is the most common skin cancer detected in Caucasians, Chinese Asians, and Japanese, the reverse was detected among Blacks and Asian Indians [32]. In the current study, there is a high incidence of skin cancers in the head and neck in comparison to other parts of the body. Sunlight is assumed to be the main environmental stress that can lead to melanomas. The head and neck are more exposed to sunlight in comparison to the trunk and other parts of the body, so they have a higher incidence of melanoma and skin cancer [33-35]. Similar to other cancers, BCC has a complex relationship with sunlight, with many lesions developing on areas that are not frequently exposed to the sun, an age-related plateau in incidence, and a significant relationship between recreational exposure and risk [35]. On the other hand, the incidence of SCC monotonically increases in the presence of sunlight [36].

The diagnosis of MF is difficult since it can affect any part of the skin and can be misdiagnosed with many skin diseases including chronic eczema, atopic dermatitis, or psoriasis in its early phase of the disease [37]. Most cases of MF have the phenotype of T-helper lymphocytes (CD3+/CD4+), while CD8+ lymphomas are relatively rare [10]. CD8+ MF is usually more aggressive than CD8- MF [11]. In the current study, 10 cases of MF were detected and most of them were CD3+/CD4; however, 6/10 cases were found to possess CD8+. These cases showed widespread lesions all over the body while the rest were localized, which agrees with the previous finding that CD8+ MF is more aggressive than CD8- MF [11]. MF was reported in Saudi Arabia in a frequent manner in Dharan (23 cases from 1995 to 2014), Jeddah (19 cases), and Madinah (14 cases from 2006 to 2017) [2,26,28].

In the current study, six cases of KS were recorded. All cases were immunocompromised due to previous HIV infections and showed positive immunostaining to human herpes virus 8. This virus was detected in all cases of KS [13,14]. The cells were found to express CD31 confirmed by positive immunostaining that suggests pluripotent cells origin of such tumors and refers to lymphatic and blood vessel differentiation. This result suggests that angiosarcoma with lymphatic differentiation could be separated as a substantial subtype from classical angiosarcoma [38,39]. One out of the six cases reported in the current study was found in the head and neck. This type of localization is common in AIDS-related KS. In contrast, it is rare in non-AIDS cases of KS [40]. It is the first time to record this type of skin tumor. However, it was reported previously in the Eastern (Dahran, 13 cases) and Western areas (Jeddah, two cases) and a single case in AlMadinah [2,26,28].

In general, DFSP was detected in 4-6% of the detected skin cancers in KSA [2,25,26,28], which matched the percentage detected in the current study (5.5%). Although DFSP possesses female predominance at 20-40 years old [41], in the current study, it was recorded in four males and only one female but within the same age range. These types of tumors are composed of fibroblastic, myofibroblastic, and histiocytic cells with eosinophilic cytoplasm that are generally positive for CD68 and negative for CD34 [42]. DFSP that showed CD34+ occurred rarely and they were found in about 5% of the DFSP [43]. CD34- DFSP could be due to the transformation of CD34-positive to CD34-negative fibroblasts/myofibroblasts [44]. In contrast, most of the DFSP tumors, in the current study, showed positive immunostaining to CD34 but negative for S100, which is suggestive of myxoid DFSP and obviates the myxoid nerve sheath tumor.

The main limitation of the current study is that many cancer cases that were referred to main oncology centers were not included in the current study. This may contribute to the relatively low number of cases. The lack of precancerous lesions and benign lesions is also another limitation of the current study.

## Conclusions

In conclusion, both SCC and BCC were the most prevalent cancers in Taif where SCC is slightly more prevalent than BCC. Melanoma was detected in a very low frequency. MF was detected in a considerable percentage with the aggressive CD8+ MF being more prevalent than CD8- MF. DFSP cancers were detected and confirmed as myxoid DFSP based on the histopathological features and CD34+ S100- immunostaining. A considerable number of KS present were all from immunocompromised HIV-positive aged patients with HHV8 and CD31 immunostaining confirmed in all cases.
